# Novel *N*-arylamide derivatives of (*S*)-perillic acid ((*S*)-PA): *in vitro* and *in vivo* cytotoxicity and antitumor evaluation†

**DOI:** 10.1039/c9ra03382c

**Published:** 2019-06-26

**Authors:** Yusif Mohammed Mukhtar, Kaili Wang, Ran Li, Wenwen Deng, Michael Adu-Frimpong, Huiyun Zhang, Kangyi Zhang, Chenlu Gu, Ximing Xu, Jiangnan Yu

**Affiliations:** Department of Pharmaceutics and Tissue Engineering, School of Pharmacy, Jiangsu University 301 Xuefu Road Zhenjiang 212001 P. R. China xmxu@ujs.edu.cn; Department of Basic and Biomedical Sciences, College of Health and Well-Being P. O. Box 9, Kintampo Ghana

## Abstract

Hepatocellular carcinoma (HC) and glioblastoma (GBA) are the most commonly aggressive malignant liver and brain tumors. Based on an established method for the synthesis of amide, two novel analogues (4 and 5) of (*S*)-perillic acid were synthesized and their structures were affirmed using nuclear magnetic resonance spectroscopic analysis. An MTT cytotoxic assay showed that our derivatives (4 and 5) demonstrated a substantial anti-proliferative effect against HC (HepG2) and GBA (U251) cell lines. Particularly, compound 5 showed growth inhibitory (IC_50_) effects on U251 (IC_50_ = 3.10 ± 0.12 μg mL^−1^) and HepG2 cells (IC_50_ = 1.49 ± 0.43 μg mL^−1^), which fall within the acceptable standard recommended by the National institute of cancer (Bethesda, MD, USA) for the selection of anticancer drug candidates. Consequently, we assessed the *in vivo* antitumor and organ/tissue toxicity of 4, 5 and 5-fluorouracil (5-FU) in hepatoma H22-inoculated mice. The results obtained indicated remarkable tumor growth inhibition with no substantial toxicological effects on the mice and the organs/tissues in the treated groups compared well with the control.

## Abstract 2

(*S*)-Perillic acid derivatives bearing *N*-aryl moiety with enhanced cytotoxic and antitumor effect against hepatocellular carcinoma and glioblastoma.

## Graphical abstract

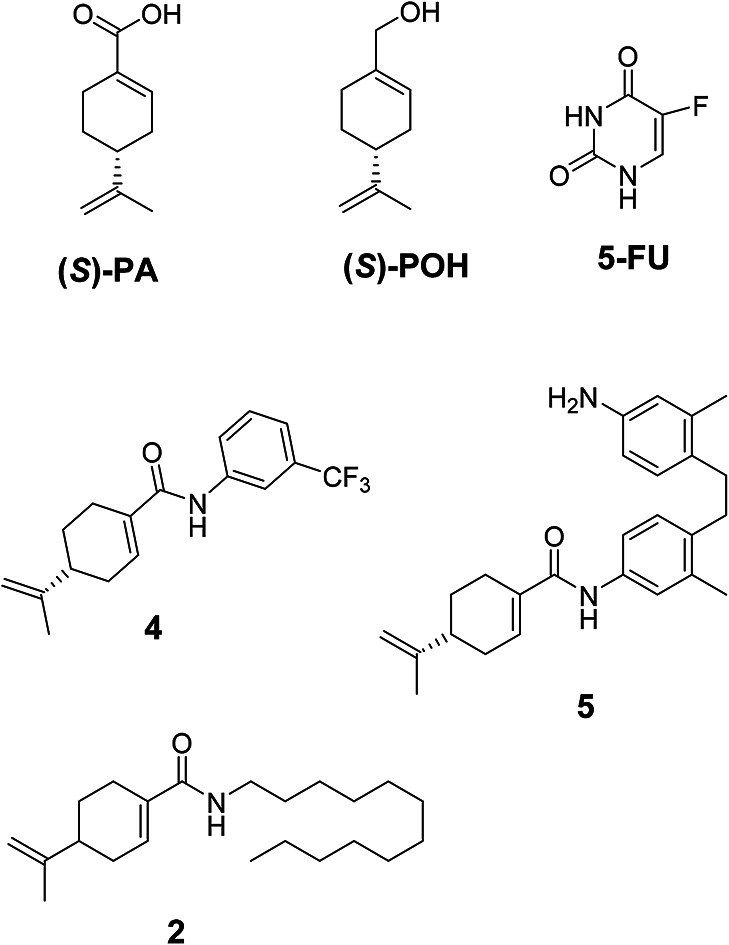

## 1 Introduction

Over the years, medicinal chemists have substantially contributed to the treatment of diseases through discovery and development of therapeutic essential drugs. The rationale for the design and synthesis of small organic molecules is to develop effective treatment options for chronic diseases. Indeed, one of the key goals of medicinal chemists is to synthesize these molecules through either simple chemical modifications or total synthesis of natural medicines. Cancer research is one of the critical fields of medicine and has attracted much interest from the scientific community around the entire globe. In view of its deleterious impact on human health, the search for novel effective agents for cancer treatment is imperative and involves a collective task for all medicinal scientists in this research area. There are three main established therapeutic approaches for cancer treatments, *viz.*, surgery, radiation and chemotherapy. However, the aforementioned treatment strategies are mostly challenged by the possible way to overcome the acquired capabilities of cancer cells.^1,2^

Hepatocellular carcinoma (HCC) and glioblastoma (GBA) are types of the most aggressive and common malignant liver and brain tumors respectively with high-tumor related mortality.^3–5^ In this regard, new treatments are currently being tested in clinical trials, albeit the tumors (GBA and HCC) exhibiting high resistant to chemotherapy.^6,7^ Notably, doxorubicin, 5-FU and cisplatin are the most effective systemic drugs for HCC whereas temozolomide is considered as the first choice chemotherapeutic agent for GBA treatment. Indeed, most of these chemotherapeutic agents have some inherent adverse effects, albeit the unmet need to discover novel small molecules with remarkable potency and less toxicity to normal tissues/organs as well as promising drug-like benefits for GBA and HCC treatment.

Monocyclic terpenoids are recognized as potential anticancer/medicinal agents.^8^ For instance, limonene and its metabolites, perillyl alcohol (POH) and perillic acid (PA) have demonstrated promising antitumor activities across the broad spectrum of cancers.^9–15^ The biochemical functions of these monoterpenoids includes but not limited to inhibition of prenylation enzymes, Geranylgeranyl transferase 1 (GGTase 1) and Farnesyl transferase (FTase),^16^ inhibition of calcium-dependent constitutive nuclear factor κB pathway,^17^ inhibition of Na^+^/K^+^-ATPase,^18^ the arrest of cell cycle at G_0_/G_1_ phase,^19^ interruption of rat sarcoma (Ras)/mitogen-stimulated kinase (MAPK)-dependent interleukin-2 (IL-2) and suppression of IL-2 plus IL-10 in mitogen activated T-lymphocytes,^20^ depletion of membrane-bound Ras protein,^20^ inhibition of thymidine incorporation,^21^ the induction of apoptosis with increasing expression of B-cell lymphoma-2 (Bcl-2)-associated X protein (Bax)^22^ and p21,^22^ as well as caspase-3 activity.^22^

The bioactivities of limonene and its metabolites support their potential chemoprotective property claim. However, compared to standard antitumor agents, these monoterpenes are considered less potent. In addition, available evidence indicated that high doses of monoterpenoids are required to elicit antitumor effects *in vivo*.^23–25^ Therefore, a design and synthesis of (*S*)-POH and (*S*)-PA analogues with improved potency and/or drug-like properties may further explain the pharmacological effects of these monocyclic terpenes in the treatments of cancer and other related illnesses. Existing data suggest that numerous studies have been conducted on the derivatives or synthetic analogues of these chemical agents.^26–28^ However, the discovery of new analogues with more bioactive potentials and especially with lower IC_50_ values (less than 4.0 μg mL^−1^, *i.e.* standard for the acceptance of a drug as an anticancer agent according to the National institute of cancer, MD, Bethesda, USA) could be clinically useful in the treatment of cancer. Therefore, this study sought to potentiate the anticancer effect of PA/POH through chemical modification and subsequent *in vitro* and *in vivo* evaluations of the cytotoxic and antitumor effects of the targeted analogues in comparison with the parent drugs and standard reference drug.

**Scheme 1:**
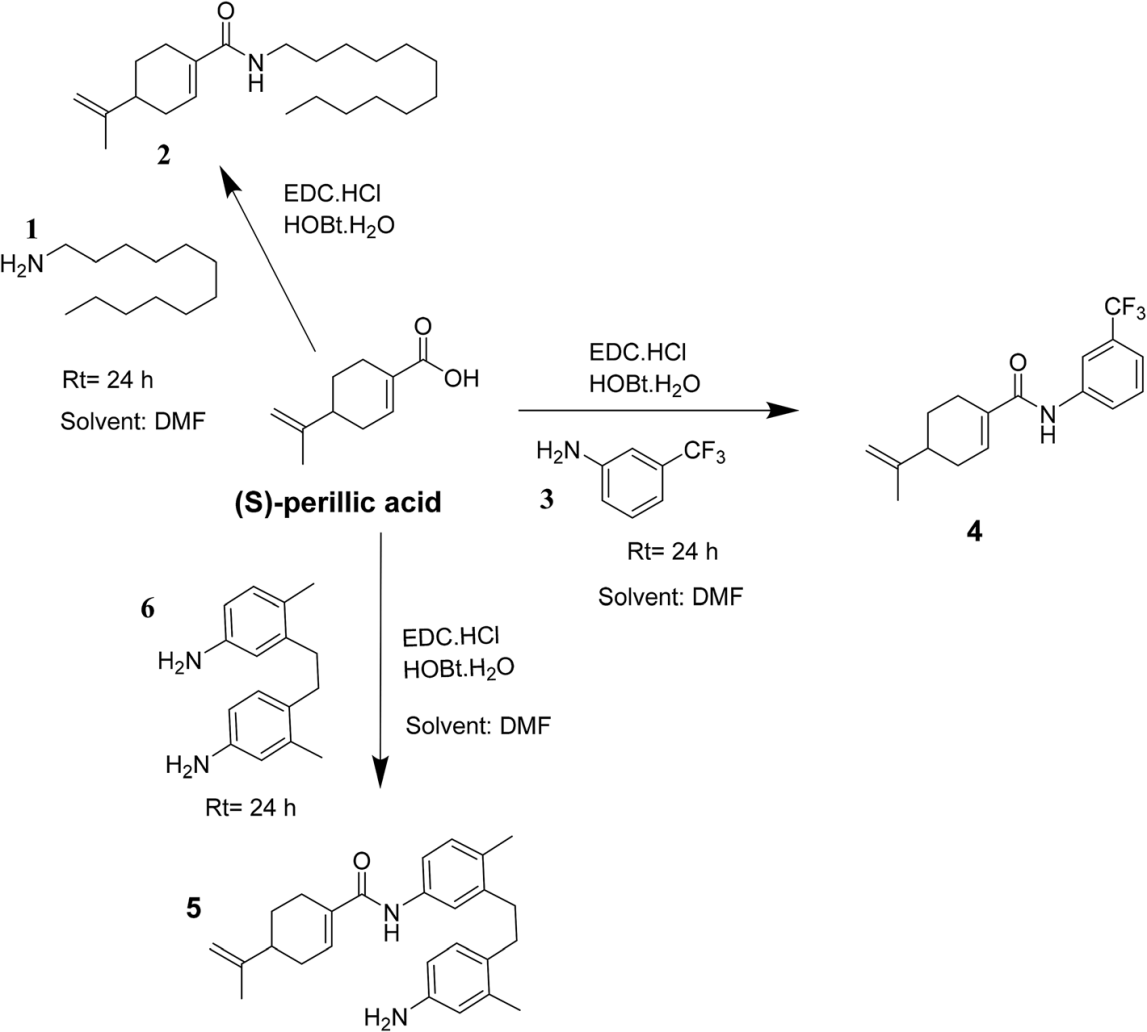
Reaction route leading to the formation of amide of (*S*)-perillic acid 2, 4 and 5.

Indeed, the design of compounds was initially inspired by the enhanced antiproliferative effect of previously reported perillyl glycoside, PG9 (ref. 27) over the parent drug (*S*)-POH. Herein, we isosterically replaced the perillyl methylene group of the abovementioned glycoside by a carbonyl functionality while arylamines was applied as substitutes for the sugar moiety (*i.e.* (*S*)-PA was coupled to the respective amine) to obtain the target compounds (4 and 5) as shown in Scheme 1. Moreover, we substituted the sugar moiety for the arylamines because previous work reported that an enhanced *in vitro* antiproliferative activity of PG9 was sugar dependent.^27^ Accordingly, we hypothesized that by this simple design, the cytotoxic effect of the targeted (*S*)-PA analogues could be improved than (*S*)-PA/POH when the antitumor properties of (*S*)-PA analogues 4 and 5 on HepG2 and U251 cell lines were screened. Also, we speculated that the attachment of aromatic side chain could enhance (*S*)-PA accumulation in tissue.^29,30^

## 2 Experimental section

### 2.1 Materials and methods

*N*,*N*-Diisopropylethylamine (DIPEA) [MACKLIN®], *N*-(3-dimethylaminopropyl)-*N*′-ethylcarbodiimide hydrochloride (EDC·HCl) [MACKLIN®], dodecylamine [MACKLIN®], 1-hydroxybenzotriazole hydrate (HOBt·*x*H_2_O) [MACKLIN®], 3-(trifluoromethyl)aniline [MACKLIN®], 4,4′-diamino-2,2′-dimethylbibenzyl [TCI], *N*,*N*′-dimethylformamide (DMF) [Shanghai Rich joint], sodium chloride (NaCl) [KESHI®], sodium hydroxide (NaOH) [KESHI®], sodium sulfate (Na_2_SO_4_) [SCR®], dichloromethane [KESHI®], ethanol [KESHI®], ethylacetate (EtOAc) [KESHI®], methanol (MeOH) [KESHI®], hexane [KESHI®], petroleum-ether [KESHI®], chloroform (CHCl_3_) [KESHI®], dimethylsulfoxide (DMSO), deuterated chloroform (CDCl_3_) [J&K®], deuterated dimethylformamide (DMSO, d6) [J&K®], (*S*)-perillic acid [Sigma Aldrich], (*S*)-perillyl alcohol (POH) [Sigma Aldrich, 96%], HepG2 human liver cancer cell lines [cell bank of academy of science, Shanghai, China], H22 murine hepatoma cancer cell lines [Shanghai Sixing Biotechnology], Foetal bovine serum [Invitrogen Co., CA, USA], Dulbecco's modified Eagle's medium (DMEM) [Invitrogen Co., CA, USA], 5-Fluorouracil (5-FU) [Aladdin® 99%], MTT [Beyotime Institute of biotechnology, Jiangsu, China], trypsin MTT [Beyotime Institute of biotechnology, Jiangsu, China], Cremophor EL® [Aladdin®].

### 2.2 Chemistry

#### 2.2.1 Synthesis and characterization

##### 2.2.1.1 Synthesis of novel amides of *S*-perillic acid (*i.e.*4 and 5)

A solution of 1.0 mmol of the acid ((*S*)-perillic acid), 0.75 mmol HOBt·*x*H_2_O and 0.75 mmol EDC·HCl was stirred for 10 min prior to the addition of 3-(trifluoromethyl)aniline (or 4,4′-diamino-2,2′-dimethylbibenzyl or dodecylamine) (1.0 mmol) in DMF (20 mL) at room temperature. Next, the reaction mixture was stirred at room temperature for 24 h. Then, water (30 mL) was added to the reaction mixture while stirring and afterwards the aqueous phase was extracted with ethyl acetate (2 × 40 mL). The combined organics were then washed with brine, dried over anhydrous Na_2_SO_4_ and concentrated under reduced pressure. The residual oil was then purified *via* silica gel column chromatography (hexane/ethyl acetate) to obtain the compounds 2, 4 and 5 in solid form at respective yields of 52.2%, 65.7% and 35.9%. The compounds were characterized using electrospray ionization mass spectrometry (ESI-MS), high-resolution mass spectrometry (HRMS) and nuclear magnetic resonance spectroscopy (^1^H-NMR, ^13^C-NMR and or ^19^F-NMR).

###### *N*-Dodecyl-4-(prop-1-en-2-yl)cyclohex-1-ene-1-carboxamide

^1^H NMR (400 MHz, CDCl_3_) *δ* 7.28 (s, 1H), 6.70–6.63 (m, 1H), 5.71 (s, 1H), 4.78 (dd, *J* = 16.0 Hz, 2H), 3.33 (dd, *J* = 12.0 Hz, 2H), 2.51–2.03 (m, 5H), 2.03–1.90 (m, 1H), 1.78 (s, 3H), 1.66–1.44 (m, 3H), 1.31 (d, *J* = 16 Hz, 18H), 0.89 (dt, *J* = 12.0 Hz, 3H). ^13^C NMR (101 MHz, CDCl_3_) *δ* 168.2, 148.9, 133.1, 132.5, 109.2, 40.2, 39.6, 31.9, 30.7, 29.7, 29.6, 29.6, 29.6, 29.5, 29.3, 29.3, 27.1, 27.0, 24.8, 22.7, 20.7, 14.1. ES-MS, [M + H]^+^ 334.69 and [M + Na]^+^ 356.68.

###### 4-(Prop-1-en-2-yl)-*N*-(3-(trifluoromethyl)phenyl)cyclohex-1-ene-1-carboxamide (4)

^1^H NMR (400 MHz, CDCl_3_) *δ* 7.87 (s, 1H), 7.80 (t, *J* = 8.0 Hz, 1H), 7.67–7.53 (m, 1H), 7.50–7.41 (m, 1H), 7.37 (d, *J* = 8.0 Hz, 1H), 7.28 (s, 1H), 6.80 (dd, *J* = 4.0 Hz, 1H), 4.80 (dd, *J* = 16.0 Hz, 2H), 2.62–2.52 (m, 1H), 2.45–2.29 (m, 2H), 2.29–2.11 (m, 2H), 2.06–1.94 (m, 1H), 1.81 (d, *J* = 12.0 Hz, 3H), 1.67–1.50 (m, 1H). ^13^C NMR (101 MHz, CDCl_3_) *δ* 166.4, 164.5, 148.4, 146.2, 138.6, 135.5, 134.4, 133.7, 133.5, 129.5, 128.6, 123.0, 122.5, 120.7, 120.7, 116.7, 116.6, 77.3, 77.2, 77.0, 76.7, 40.0, 30.9, 27.0, 24.7, 20.7. ^19^F NMR (376 MHz, CDCl_3_) *δ* −56.69 to −78.96 (m). HRMS: calculated for calculated for [C_17_H_19_F_3_NO + H]^+^ 310.1419, found 310.1411.

###### *N*-(4-(4-Amino-2-methylphenethyl)-3-methylphenyl)-4-(prop-1-en-2-yl)cyclohex-1-ene-1-carboxamide (5)

^1^H NMR (400 MHz, CDCl3) *δ* 7.40 (d, *J* = 2.0 Hz, 1H), 7.35 (s, 1H), 7.32 (dd, *J* = 8.0, 2.2 Hz, 1H), 7.28 (s, 1H), 7.10 (d, *J* = 8.0 Hz, 1H), 6.93 (d, *J* = 8.0 Hz, 1H), 6.79–6.74 (m, 1H), 6.58–6.48 (m, 2H), 4.80 (dd, *J* = 16.0 Hz, 2H), 2.83–2.70 (m, 4H), 2.56 (dd, *J* = 16.0 Hz, 1H), 2.47–2.28 (m, 5H), 2.28–2.09 (m, 5H), 2.04–1.94 (m, 1H), 1.80 (s, 3H), 1.65–1.49 (m, 1H), 1.41–1.18 (m, 2H). ^13^C NMR (101 MHz, CDCl_3_) *δ* 166.3, 165.7, 148.7, 144.6, 144.4, 136.8, 136.8, 136.7, 136.6, 136.5, 136.1, 135.8, 134.4, 133.8, 133.6, 133.4, 133.1, 130.3, 130.1, 129.8, 129.6, 129.4, 122.7, 121.7, 119.0, 117.7, 117.3, 117.2, 116. 8, 112.9, 112.0, 109. 3, 77.3, 77.2, 77.0, 76.7, 40.15, 34.1, 33.7, 33.4, 32.4, 25.1, 24.9, 22.0, 20.8, 19.6, 19.4, 19.4, 18.4. HRMS: calculated for [C_26_H_33_N_2_O + H]^+^ 389.2593, found 389.2577.

### 2.3 Biological experiments

#### 2.3.1 Cell culturing and pilot experiments

Human cancer cells (HepG2 and U251) were grown in Dulbecco's Modified Eagle's Medium supplemented with 10% fetal bovine serum, 1% streptomycin and penicillin prior to incubation for 24 h at 37 °C in a 5% CO_2_ to allow the cells to grow until the plate was 80% confluent. Briefly, the cells were dislodged *via* trypsinization and then (about 1.0 × 10^3^ to 1 × 10^4^ per cells) plated into the 96-well plate and cultured in a humidified 5% CO_2_ at 37 °C for 24 h. After medium removal, aliquot (100 μL) of fresh medium containing a single dose (100 μg mL^−1^ in DMSO) concentration of test compounds (*i.e.* 5-FU, *S*-PA, 2, 4 and 5) were added into the plates. The plates were incubated for 48 h under standard conditions, *viz.*, 5% CO_2_ and 37 °C in a humidity control incubator. At the end of incubation, 20 μL of MTT (5 mg mL^−1^) was added to each well. The plates were incubated for an additional 4 h at standard culture conditions. Subsequently, MTT was removed and DMSO (100 μL) was added prior to shaking for 5–10 s. Afterwards, the fluorescence of MTT was measured for each well at 570 nm (BIV-TEK INSTRUMENTS INC). The cells were incubated in culture medium with DMSO serving as a control for cell viability determination. Each experiment was repeated in triplicate. The effect of test compounds against the viability of cells was evaluated following this mathematical equation: percentage viable cell = AbsT570/AbsC570; where AbsT570 = relative absorbance of test compound@570 nm and AbsC570 = relative absorbance of blank/control@570 nm.

#### 2.3.2 Cytotoxic assay

The cytotoxic effects of the tested compounds on human glioblastoma (U251), hepatocellular carcinoma (HepG2) and normal fibroblast (3T6) cell lines were determined using MTT assay. The cells (1.0 × 10^3^) were seeded in 96-well plate for 24 h. The culture medium was replaced with 100 μL of media containing (0, 5, 10, 15, 20, 25 and 30 μg mL^−1^) concentrations of compounds 4, 5 and POH, as well as 5-FU (0, 10, 50 and 100 μg mL^−1^). Then, the cells were exposed to a humidified 5% CO_2_ at 37 °C for 24, 48 and 72 h. Next, the treated medium of each well was substituted with fresh medium (100 μL) and MTT (5 mg mL^−1^, 20 μL). The plates were then incubated for 4 h under the aforementioned conditions. Subsequently, MTT was removed and DMSO (100 μL) was added followed by shaking for 5–10 s. Measurement of MTT fluorescence was conducted for each well at 570 nm (BIV-TEK INSTRUMENTS INC). The cells were incubated in the culture medium with DMSO as a control for cell viability estimation. Each experiment was triplicated.

### 2.4 Animal experiments

The Laboratory Animal Center of Jiangsu University supplied forty (40) Kunming mice (males, weight varying between 20 and 25 g). Animal Ethics and Experimentation Committee of Jiangsu University approved the experimental procedures according to the requirements for the Prevention of Cruelty to Animals Act 1986 and they complied with the National Institute of Health Guide for Care and Use of Laboratory Animals [No. SCXK (Su) 2018-0053]. The animals were housed for 14 days to adapt to standard experimental conditions at temperature (25 ± 2 °C), relative humidity (56 ± 5%) alongside a 12/12 h light/dark cycle with unrestricted access to rodent diet and water before the experiments.

#### 2.4.1 *In vivo* antitumor and toxicity evaluation

Hepatoma carcinoma (H22) cells were cultured in medium under suitable conditions. The cells (about 5.0 × 10^6^ per cells) were injected intraperitoneally into the mice. After seven days (when the cells were richly grown), the mice were sacrificed and the cells were harvested. Subsequently, the collected cells were centrifuged and the supernatant discarded, prior to thrice washing with PBS. The resulting H22 cells were dissolved in PBS before injection. Further, one million cells (in a volume of 100 μL) of the H22 cell suspension was injected subcutaneously into the right flank of each mouse. After 3 days of inoculations (when tumor size reached 130 mm^3^), the mice were divided into 6 groups (*n* = 6) *viz.*, vehicle control of ethanol-cremorphor EL®-saline (ECS), 30 mg kg^−1^ & 60 mg kg^−1^ for compound 4, 15 mg kg^−1^ & 30 mg kg^−1^ for 5 and 25 mg kg^−1^ for 5-FU. The mice were fasted overnight before and 2 h after treatment, but were allowed unrestricted access to water. The mice were subjected to treatments for 28 days prior to sacrifice, while the size of tumor, liver, spleen and kidney as well as body weights were recorded. Notably, the vehicle was prepared as reported elsewhere^31^ with slight compositional changes.

#### 2.4.2 Histopathological analysis

Following fixation in 4% formalin, the tumor, liver, kidney and spleen were cut into small pieces and embedded in paraffin. Next, 5 μm-thick histological sections were obtained and stained in hematoxylin and eosin (H&E). Histological analysis was carried out using light microscopy to assess the possible damages caused by the treatment.

#### 2.4.3 Statistical analysis

Data collected were presented as mean ± SD/SEM and the differences among the experimental groups were evaluated using one-way/two-way analysis of variance (ANOVA) where appropriate followed by Tukey's multi-comparison test. Statistical significance level was considered at *p* < 0.05. All the statistical analyses were performed using GraphPad software (intuitive software for sciences, San Diego, CA, USA).

## 3 Results and discussions

### 3.1 Chemistry

**Fig. 1:**
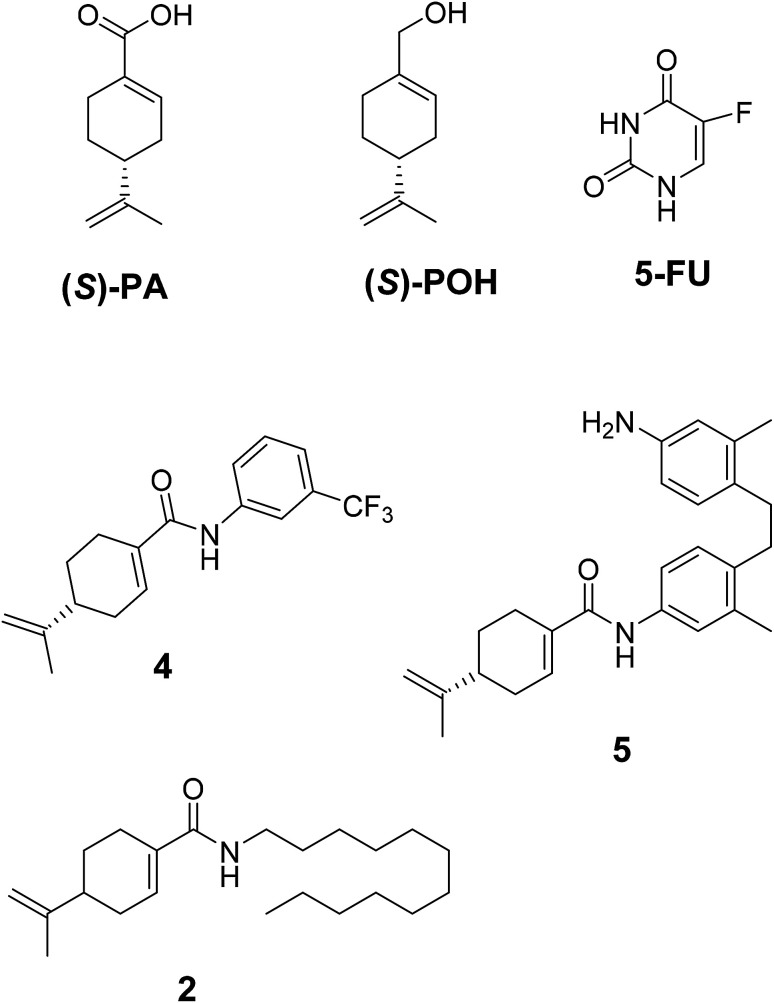
Synthesized and reference compounds used in this study.

The compounds 2, 4 and 5 were synthesized through a general coupling reaction between a carboxylic acid and amine in the presences of a coupling agent, EDC·HCl (*N*-(3-dimethylaminopropyl)-*N*′-ethylcarbodiimide hydrochloride) and an activator HOBt·H_2_O (1-hydroxybenzotriazole hydrate) (Scheme 1). After synthesis and purification, the targeted compounds (Fig. 1) were structurally characterized *via* spectroscopic analysis. The spectroscopic techniques employed in this study were ESI-MS and or HRMS and NMR, *i.e.*^1^H, ^13^C and ^19^F. The detailed structural information of the compounds 2, 4 and 5 has been attached to the experimental section while the spectra data are presented in the ESI.† The (*S*)-PA, (*S*)-POH and 5-FU were obtained from commercial sources and were directly used without any treatment. However, the purities (>90%) of the synthesized compounds were analyzed with Schimadzu RP-HPLC system with C_18_ analytical column prior to the biological assays.

### 3.2 Biological assays

**Fig. 2:**
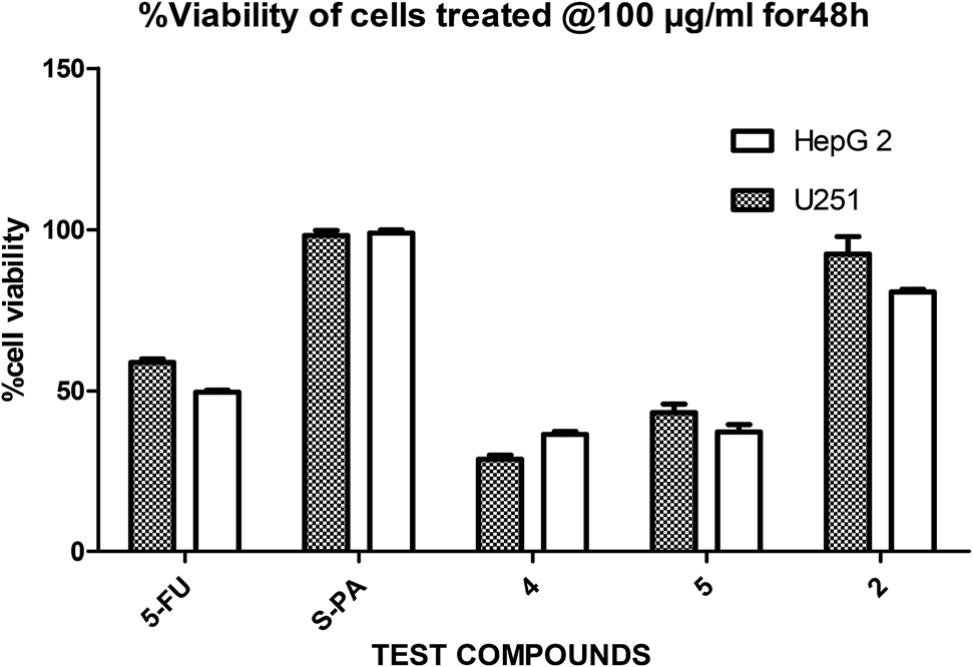
Single dose (100 μg mL^−1^) comparison of agents 2, 4, 5, 5-FU and *S*-PA against HepG2 and U251 cell lines.

In a pilot experiment, the anticancer effects of (*S*)-PA and its synthetic analogues 2, 4 and 5 on HepG2 and glioma, U251 cells were assessed *via* percentage viable cell to vehicle control at a single dose of 100 μg mL^−1^ using MTT cytotoxic assay approach. From the obtained result (Fig. 2), the parent compound *i.e.* (*S*)-PA did not show any substantial anticancer effect compared with its novel analogues (4 and 5) and 5-FU. Also, compound 2, an alkylamide conjugate of (*S*)-PA showed low anticancer potential compared with agents 4, 5 and 5-FU. Based on these results, compounds (*i.e.* 5-FU, 4, and 5) with more than 45% inhibitory effect in both U251 and HepG2 cell lines (based on single dose analysis) were further subjected to a multiple dose study for IC_50_ measurement with POH (a notable potent monocyclic monoterpene) as control.

**Fig. 3:**
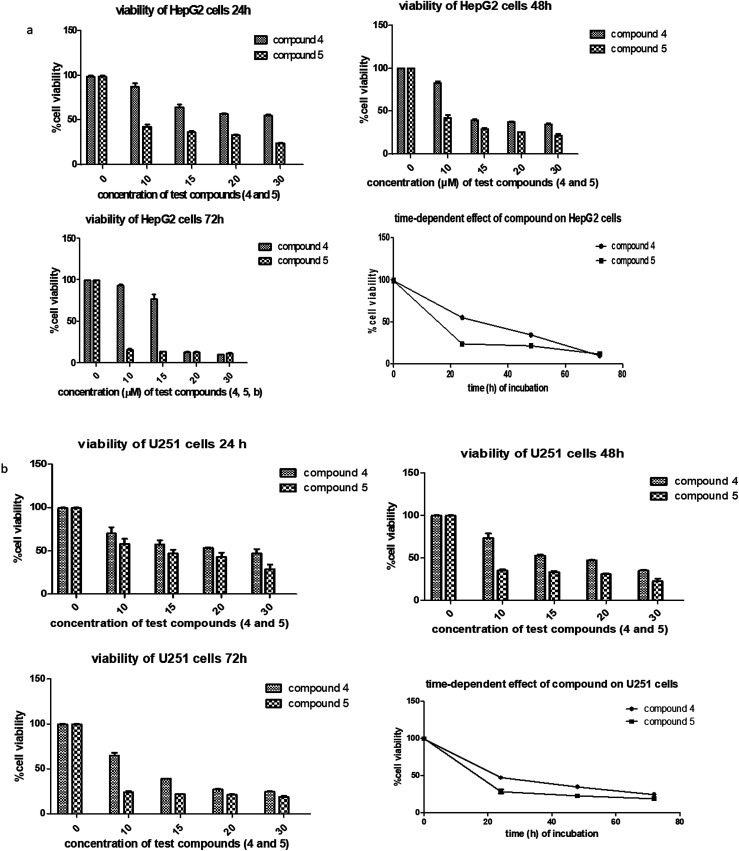
(a) Dose-and time dependency effects of compounds 4 and 5 against HepG2 cell lines. The cells were treated in concentration between 10–30 μg mL^−1^. The time dependency study was measured at a single concentration of 30.0 μg mL^−1^ at 24, 48, and 72 h. Two-way ANOVA accompanied by Tukey's multiple comparison test disclose the significance effect of each compound on the HepG cell line. Graphs are shown as the mean ± SD, a representative of three independent experiments. (b) Dose and time dependency effects of compounds 4 and 5 against U251 cell line. The cells were treated in concentration between 10–30 μg mL^−1^. The time dependency study was measured at a single concentration of 30.0 μg mL^−1^ at 24, 48, and 72 h. Two-way ANOVA accompanied by Tukey's multiple comparison test disclose the significance effect of each compound on the U251 cell line. Graphs are shown as the mean ± SD, a representative of three independent experiments.

Next, the inhibitory effects of the compounds (POH, 5-FU, 4 and 5 at different doses) were evaluated in U251 glioblastoma, HepG2 hepatocellular carcinoma and 3T6 normal fibroblast cells using MTT assay. As shown in Table 1, the two synthetic derivatives of (*S*)-PA demonstrated potent anticancer effect against the two cancerous cells U251 and HepG2 compared with (*S*)-POH. Besides, derivatives 4 and 5 exhibited higher cytotoxicities potentials than the 5-FU (a standard anticancer agent) against HepG2 cells as illustrated in Table 1. Moreover, the derivatives 4 and 5 inhibited the growth of the cells in time- and dose-dependent manner (Fig. 3a and b).

**Table 1:** *In vitro* cytotoxicity evaluation of agents 4, 5, 5-FU, and POH against human hepatocellular carcinoma, HepG2 cell line, human glioblastoma, and stabilizing murine fibroblast cell line 3T6^a^

Agents ID	U251, IC_50_ (μg mL^−1^)	HepG2, IC_50_ (μg mL^−1^)	3T6, IC_50_ (μg mL^−1^)
POH	110.07 ± 0.15	764.00 ± 0.10	65.29 ± 0.32
5-FU	2.38 ± 0.37	36.72 ± 7.40	2.79 ± 0.53
4	9.41 ± 0.38	18.07 ± 0.10	8.12 ± 0.11
5	3.10 ± 0.12	1.49 ± 0.43	11.59 ± 0.54

a IC_50_ assessed by the normal routine MTT assay after 72 h of incubation. Each IC_50_ value is presented as mean (IC_50_ ± SD) of three independent experiments ran in triplicate.

Likewise, the growth inhibitory effects of the tested compounds were measured on a normal fibroblast cell (3T6). The ranked cytotoxicity after 72 h of exposure was 5-FU, 4, 5 and POH (Table 1). In agreement with many anticancer drugs, derivatives 4 and 5 affected the normal cells at concentration from 20 μg mL^−1^ and beyond. Nevertheless, the derivatives were more cytotoxic to the cancer cells employed in this study than the normal 3T6 cells (Table 1).

Generally, agents recording IC_50_ values below or equal to 4.0 μg mL^−1^ are regarded as anticancer candidate according to the National cancer institute (Bethesda, MD, USA) in its selection program of anticancer drugs. As indicated in Table 1, compound 5 (IC_50_ = 3.10 ± 0.12 and 1.49 ± 0.43 against U251 and HepG2 respectively) satisfies the requirement of an anticancer drug candidate. Therefore, it was imperative to evaluate its *in vivo* antitumor potential to provide a firm background for its further developments. The effects of agents 4, 5 and 5-FU in hepatoma H22-inoculated tumor xenograft mice were evaluated and the results are illustrated in Table 2. The mean tumor weight of ethanolic cremophor EL®-saline negative control (ECS) was 2.54 ± 0.50 g. The intraperitoneal treated (T) groups comprising of agents, namely 4 (30 mg kg^−1^), 4 (60 mg kg^−1^), 5 (15 mg kg^−1^), 5 (30 mg kg^−1^), and 25 mg kg^−1^ 5-FU had an average tumor weight of 1.54 ± 0.49 g, 1.01 ± 0.27 g, 1.24 ± 0.25 g, 0.67 ± 0.17 g and 0.65 ± 0.45 g respectively. In line with the tumor weights, the inhibitory growth rates (% IGR) for the treated groups were calculated using the following formula [(ECS-T)/ECS × 100], which were computed to be 39.37% (for 30 mg kg^−1^ of agent 4), 60.24% (for 60 mg kg^−1^ of agent 4), 51.18% (for 15 mg kg^−1^ of agent 5), 73.62% (for 30 mg kg^−1^ of agent 5) and 74.41% (for 25 mg kg^−1^ of 5-FU). Indeed, the derivatives 4 and 5 showed antitumor activity at their respective doses with the highest inhibition occurring at the higher maximum doses (*i.e.* 60 mg kg^−1^ for 4 and 30 mg kg^−1^ for 5).

**Table 2:** The effect of agents 4, 5 and 5-FU on the body and organ weight of mice inoculated with H22 tumor cells^a^

Group	Body (g)	Liver (g)	Kidney (g)	Spleen (g)	Tumor (g)
ECS	41.5 ± 1.15	2.36 ± 07	0.42 ± 0.011	0.09 ± 0.008	2.54 ± 0.26
5-FU (25 mg kg^−1^)	39.5 ± 0.76	2.01 ± 0.15	0.42 ± 0.010	0.09 ± 0.008	0.65 ± 0.18***
4 (30 mg kg^−1^)	41.17 ± 1.68	2.21 ± 0.11	0.42 ± 0.013	0.10 ± 0.004	1.54 ± 0.20**
4 (60 mg kg^−1^)	41.00 ± 1.033	2.13 ± 0.11	0.38 ± 0.017	0.10 ± 0.013	1.01 ± 0.11***
5 (15 mg kg^−1^)	40.33 ± 0.84	2.20 ± 0.08	0.42 ± 0.017	0.11 ± 0.008	1.24 ± 0.10***
5 (30 mg kg^−1^)	40.17 ± 0.79	2.16 ± 0.08	0.41 ± 0.010	0.11 ± 0.011	0.67 ± 0.07***

a Data are presented as mean ± SEM (*n* = 6 mice per group with tumor H22). *p < 0.05 for all of the treated mice compared with the negative control using one-way analysis of variance (ANOVA), followed by the Tukey's multiple comparison test.

**Fig. 4:**
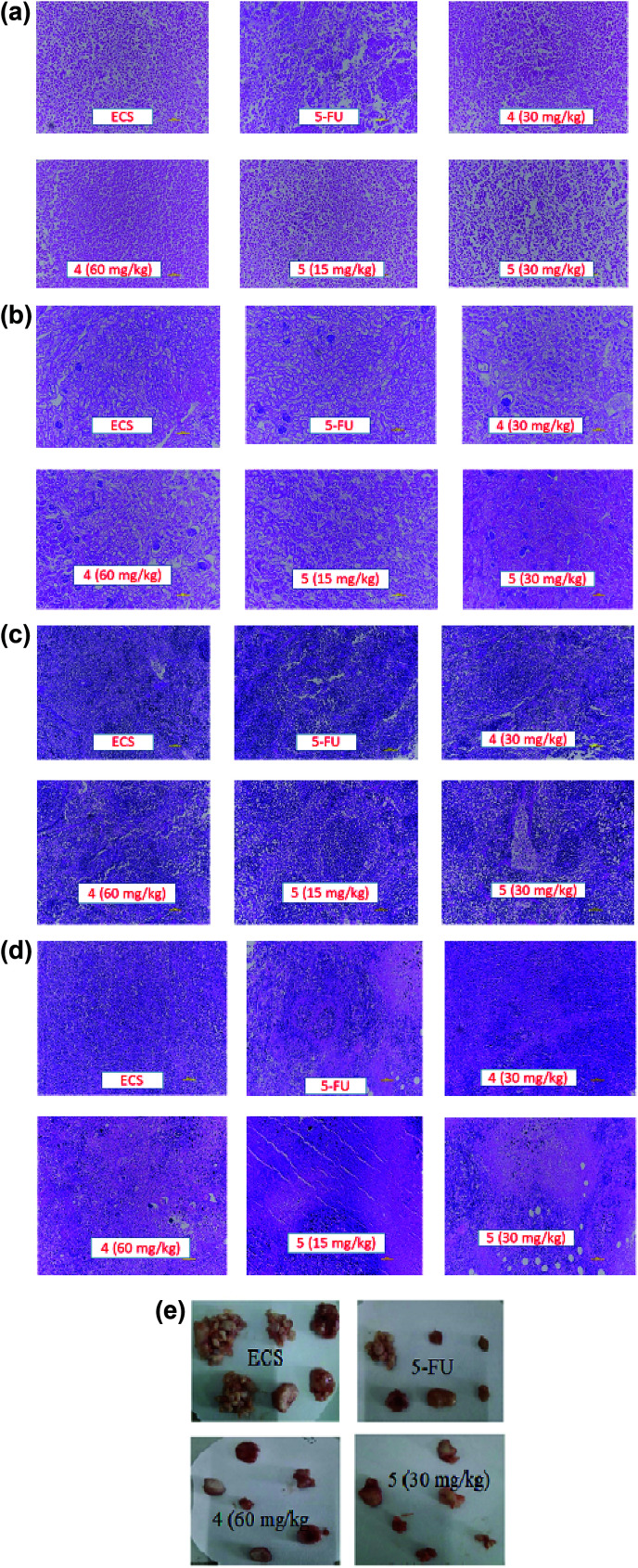
(a) The effects of derivatives 4 and 5 on livers of tumor bearing mice. Morphology was analyzed by microscopy after stained with hematoxylin–eosin. Photos from liver represent vehicle control (ECS); 25 mg kg^−1^ 5-FU; 30 mg kg^−1^ derivative 4; 60 mg kg^−1^ derivative 4; 15 mg kg^−1^ derivative 5; and 30 mg kg^−1^ derivative 5. (b) The effects of derivatives 4 and 5 on kidneys of tumor bearing mice. Morphology was analyzed by microscopy after staining with hematoxylin–eosin. Photos from kidney represent vehicle control (ECS); 25 mg kg^−1^ 5-FU; 30 mg kg^−1^ derivative 4; 60 mg kg^−1^ derivative 4; 15 mg kg^−1^ derivative 5; and 30 mg kg^−1^ derivative 5. (c) The effects of derivatives 4 and 5 on spleens of tumor bearing mice. Morphology was analyzed by microscopy after stained with hematoxylin–eosin. Photos from spleen represent vehicle control (ECS); 25 mg kg^−1^ 5-FU; 30 mg kg^−1^ derivative 4; 60 mg kg^−1^ derivative 4; 15 mg kg^−1^ derivative 5; and 30 mg kg^−1^ derivative 5. (d) The effects of derivatives 4 and 5 on tumors of tumor bearing mice. Morphology was analyzed by microscopy after stained with hematoxylin–eosin. Photos from tumor represent vehicle control (ECS); 25 mg kg^−1^ 5-FU; 30 mg kg^−1^ derivative 4; 60 mg kg^−1^ compound 4; 15 mg kg^−1^ compound 5; and 30 mg kg^−1^ compound 5. (e) The effects of derivatives 4 and 5 on tumor size of treated mice. Photos represent vehicle control group (ECS); 25 mg kg^−1^ 5-FU; 60 mg kg^−1^ derivative 4; and 30 mg kg^−1^ derivative 5.

Apart from their antitumor effects, most chemotherapeutic agents are known to adversely affect the normal organs. Therefore, the interest of this report lies in investigating the possible toxicities of the agents by identifying changes in the morphological features of livers, kidneys, spleens and tumors of the treated groups compared with the ECS group (Fig. 4a–d). Histopathological analysis of the aforementioned organs revealed no substantial morphological variations in the organs of treated groups compared with the ECS. In addition, the organ weights of the treated groups were statistically not different from the ECS (Table 2). Therefore, this result suggests that derivatives 4 and 5 might not have altered the body weights and organs, which corroborates previous antitumor activity findings.^32,33^ This is because the *in vivo* activity of most anticancer drugs are usually accompanied by damages to tissues/organs with signs like vomiting and nausea.^32^

Advancement in cancer research has led to the approval of over 100 drugs by FDA for treatment of specific cancers. Despite this improvement, several individuals diagnosed with cancer have short mean lifespan owing to the adverse effects of the supposed effective treatment options. Therefore, there is urgent need to unearth novel, safe and effective therapeutic approaches.

The effectiveness of anticancer activity of monocyclic monoterpenoids has been demonstrated in various reports.^10,22,34^ Among these monoterpenoids, (*S*)-POH is well known for its effects and has gone through phase I and II clinical trials.^35–38^ However, growing evidence indicates that higher dose of (*S*)-POH is needed to elicit its pharmacological effects.^23–25^ Therefore, the discovery of synthetic derivatives of either (*S*)-POH or its major human plasma metabolite, (*S*)-PA with enhanced activity and low dose requirement for effective antitumor activity could be rewarding in the clinical settings. In comparison with conjugates 4 and 5, (*S*)-POH recorded low cytotoxic effects in the cancer cells (HepG2 and U251) and the normal cell line (Table 1). This results support the assertion that chemical modification could enhance the biological effect of medicinal agents.^39,40^

Besides, by comparing the IC_50_ of POH and the synthetic analogs, structure–activity relationships revealed that the substitution of methylene-hydroxyl group of POH with an *N*-arylamide moiety significantly improved the cytotoxic activity than the free hydroxyl group of POH (Table 3). Likewise, the carboxylic moiety of PA showed no significant cytotoxic activity based on the preliminary experimental results (Fig. 2). Moreover, the aliphatic amide conjugate of compound 2 showed insignificant inhibitory effect on the HepG2 and U251. This observation suggests that substitution of *N*-arylamide moiety for the abovementioned functional groups on the parent PA/POH might account for the enhanced cytotoxicity of derivatives 4 and 5 against HepG2 and U251 cell lines. However, the differential inhibitory properties of agents 4 and 5 could be attributed to the additional contributing effect of the free amino group on derivative 5. This is because free amino groups have been shown to be responsible for the substantial anticancer effect of doxorubicin.^41^ Collectively, derivative 5 could serve as a potential anticancer drug candidate for the treatment of GBA and HCC based on the acceptable standard for the selection of anticancer drug candidate by National institute of cancer (Bethesda, MDA, USA), which was worth further exploration. Prompted by the *in vitro* experimental results, the antitumor effects of derivatives 4 and 5 were subsequently evaluated *in vivo* in comparison with 5-FU using murine hepatoma carcinoma H22-inoculated Kunming mice.

**Table 3:** Structure–activity relationships of derivatives 2, 4, 5, and parent compounds (*S*)-POH and (*S*)-PA against U251 and HepG2 cell lines at 72 h of administration^a^

Compound	Structure	IC_50_ (U251 cell line)	IC_50_ (HepG2 cell line)
(*S*)-PA	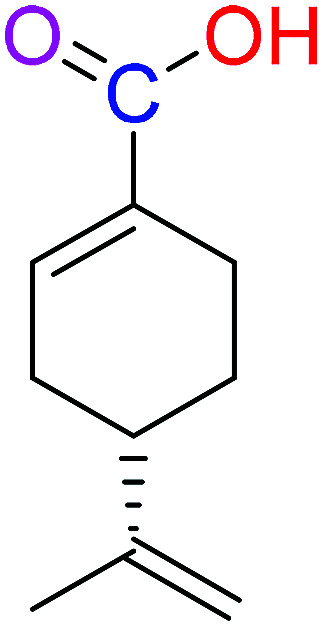	NSA	NSA
(*S*)-POH	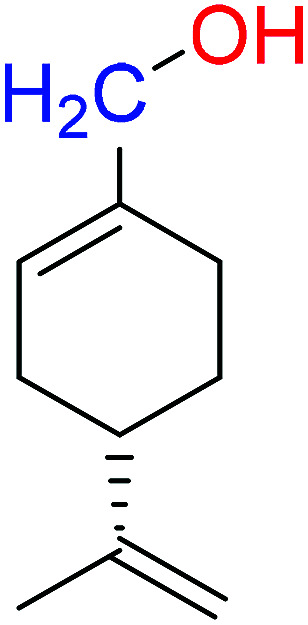	110.07 ± 0.15	764.00 ± 0.10
2	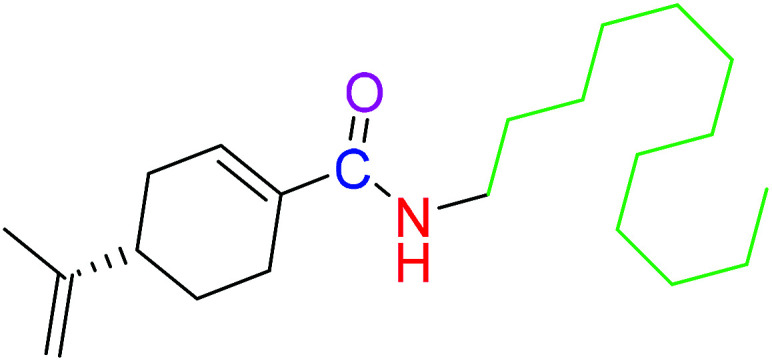	NSA	NSA
4	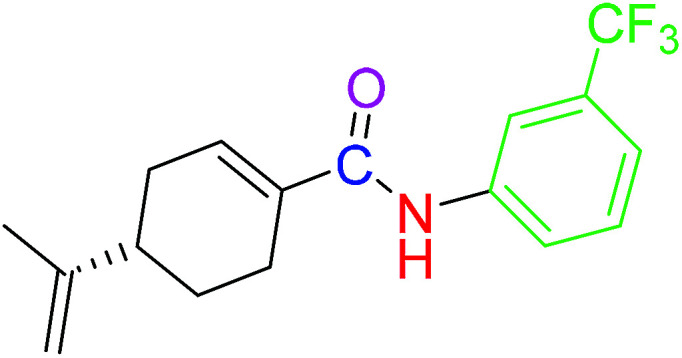	9.41 ± 0.38	18.07 ± 0.10
5	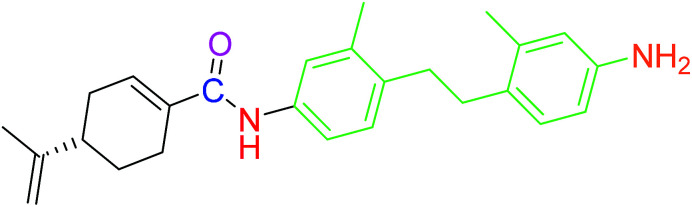	3.10 ± 0.12	1.49 ± 0.43

a NSA: no significant activity detected.

Following *in vivo* antitumor activity, derivative 5 demonstrated substantial tumor growth inhibition (at 30 mg kg^−1^), which was almost the same as that of 5-FU (at 25 mg kg^−1^). Comparatively, the observed *in vivo* activity of derivative 5 seems to be better than the perillaldehyde 8,9-epoxide (regarded as the most potent derivative of POH) in terms of the therapeutic dose.^42^ As reported by Andrade *et al.*, perillaldehyde 8,9-epoxide exhibited significant antitumor activity at a higher doses (100/200 mg per kg per day) in comparison with our derivatives. More importantly, histopathological analysis revealed no toxicity to liver, spleen and kidney of mice treated with the derivatives 4 and 5. The lack of toxicity of these agents to normal organs *in vivo* disagrees with the *in vitro* cytotoxic effect on 3T6 cell, which could be due to the sensitivity of these novel agents to 3T6 cells in the growing state.^43^ For detail understand of this phenomenon, not-too-distant future works will comprehensively investigate the possible toxicity of the derivatives using established toxicity testing models such as peripheral blood mononuclear cells (PBMCs). Also, the mechanism underlying the *in vitro* antiproliferative effects of derivatives 4 and 5 on the normal cells and their detail cytotoxicity evaluations in rodents will also be explored.

## 4 Conclusion

In conclusion, two new synthetic analogues of (*S*)-perillic acid (namely 4 and 5) were found to exhibit substantial *in vitro* cytotoxic effects on HepG2 and U251 cell lines compared with POH and 5-FU. The derivatives (4 and 5) showed cytotoxic effects in time- and dose-dependent manner. Structure–activity relationship investigation indicated that substitution of the methylene-hydroxyl functionality of either (*S*)-POH or the carboxyl functional group of (*S*)-PA with carboxamide bearing aromatic moiety successfully enhanced the anticancer activity. Further, the derivatives (4 and 5) inhibited tumor growth in hepatoma H22-inoculated mice with insignificant damage to organs/tissues compared with the control. Indeed, cancer growth inhibitory effect of derivative 5 was stronger than the parent drugs POH and PA. Based on the current findings, further studies involving derivative 5 and other structurally related compounds and or reference agents will be conducted in other cancers with the detailed cytotoxic mechanism explored.

## Conflicts of interest

There are no competing interests related to the manuscript.

## Supplementary Material

RA-009-C9RA03382C-s001
