# Supplementary material for: Novel N-arylamide derivatives of (S)-perillic acid ((S)-PA): in vitro and in vivo cytotoxicity and antitumor evaluation
Source: RSC Adv. 2019 Jun 26;9(35):19973–82. doi: 10.1039/c9ra03382c (PMC9065563; doi:10.1039/c9ra03382c)
Supplement: RA-009-C9RA03382C-s001 [file RA-009-C9RA03382C-s001.pdf]

Electronic Supplementary Material (ESI) for RSC Advances.
This journal is © The Royal Society of Chemistry 2019

SPECTROSCOPIC AND HPLC ANALYSIS OF COMPOUNDS

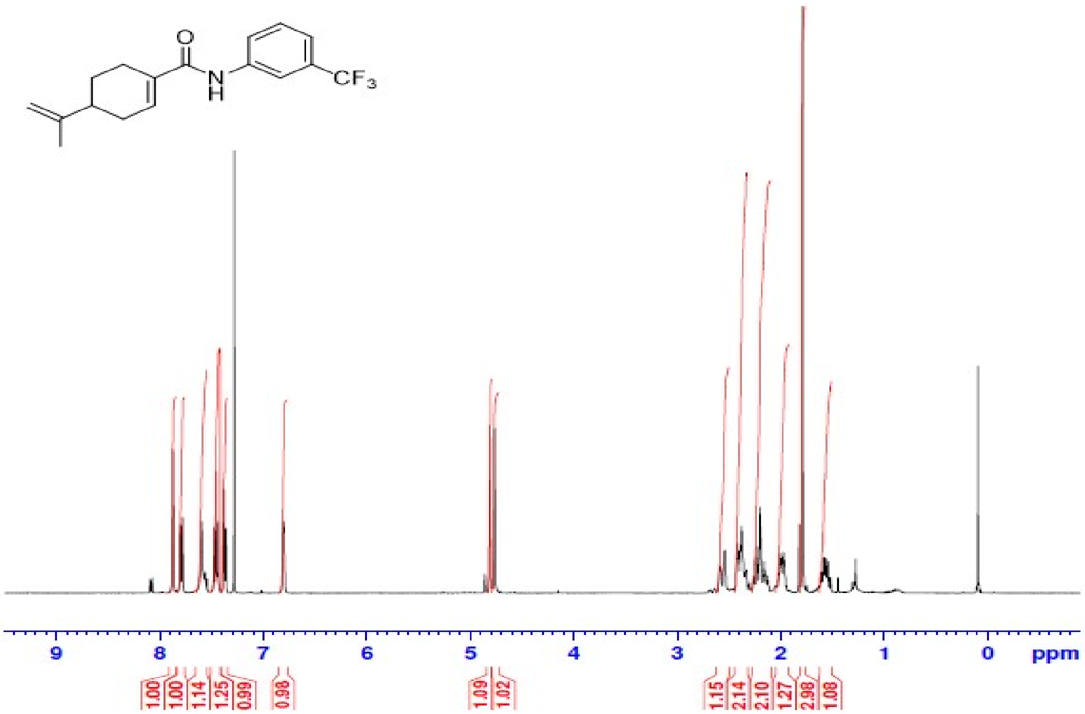

Figure S1 ^{1}HNMR spectrum of 4-(prop-1-en-2-yl)-N-(3-(trifluoromethyl)phenyl)cyclohex-1-ene-1-carboxamide (**4**) obtained on NMR spectrometer (Bruker 400 Ultra Shield™)

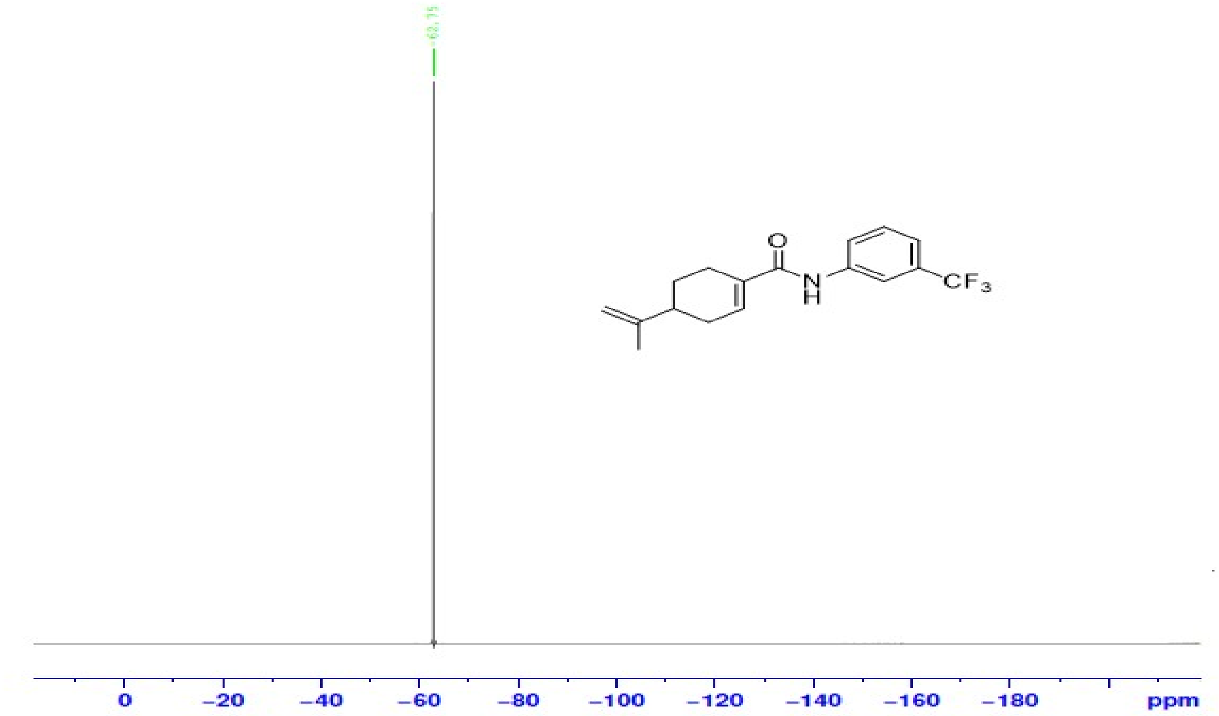

Figure S2 ^{19}F NMR spectrum of 4-(prop-1-en-2-yl)-N-(3-(trifluoromethyl)phenyl)cyclohex-1-ene-1-carboxamide (**4**) obtained on NMR spectrometer (Bruker 400 Ultra Shield[TM])

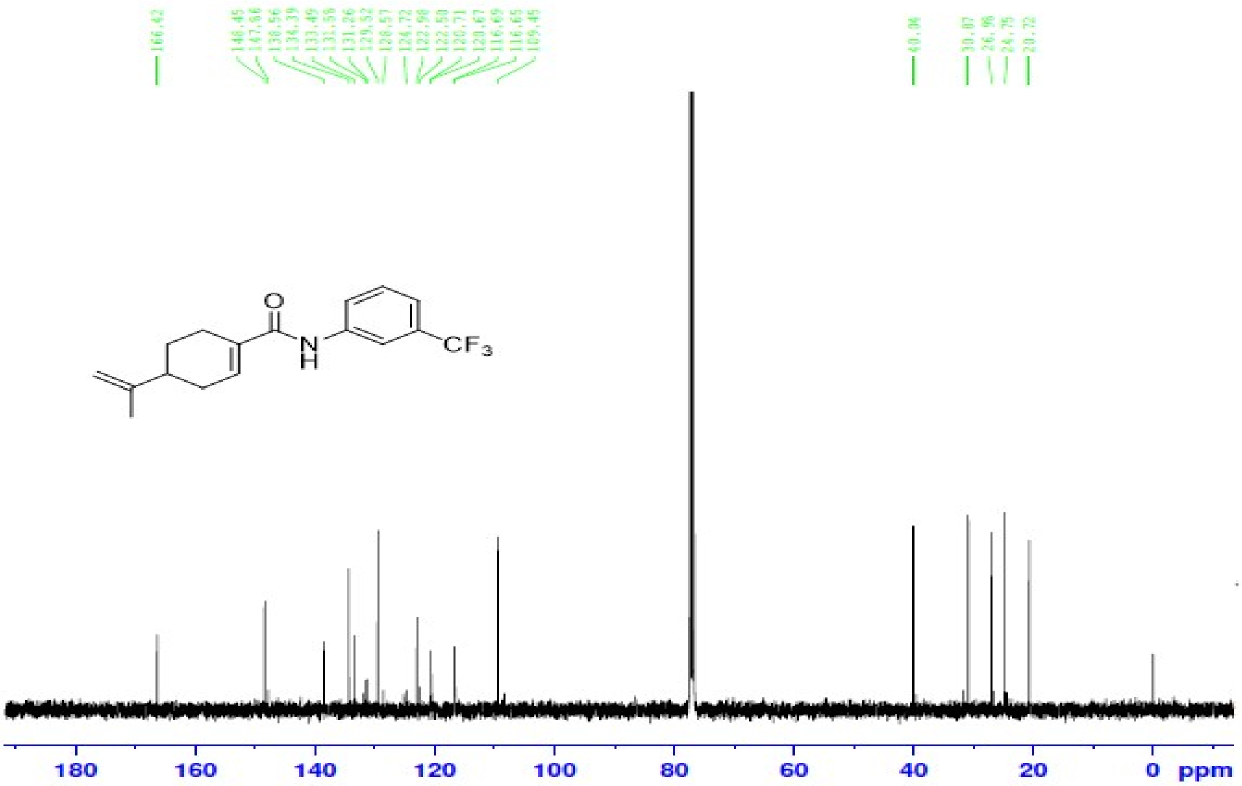

Figure S3 ^{13}C NMR spectrum of 4-(prop-1-en-2-yl)-N-(3-(trifluoromethyl)phenyl)cyclohex-1-ene-1-carboxamide (**4**) obtained on NMR spectrometer (Bruker 400 Ultra Shield™)

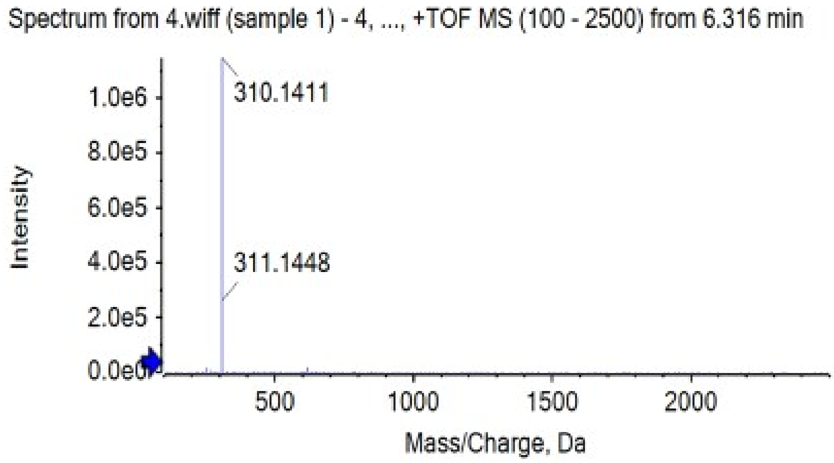

Figure S4 HRMS of 4-(prop-1-en-2-yl)-N-(3-(trifluoromethyl)phenyl)cyclohex-1-ene-1-carboxamide (**4**), [M+H] peak

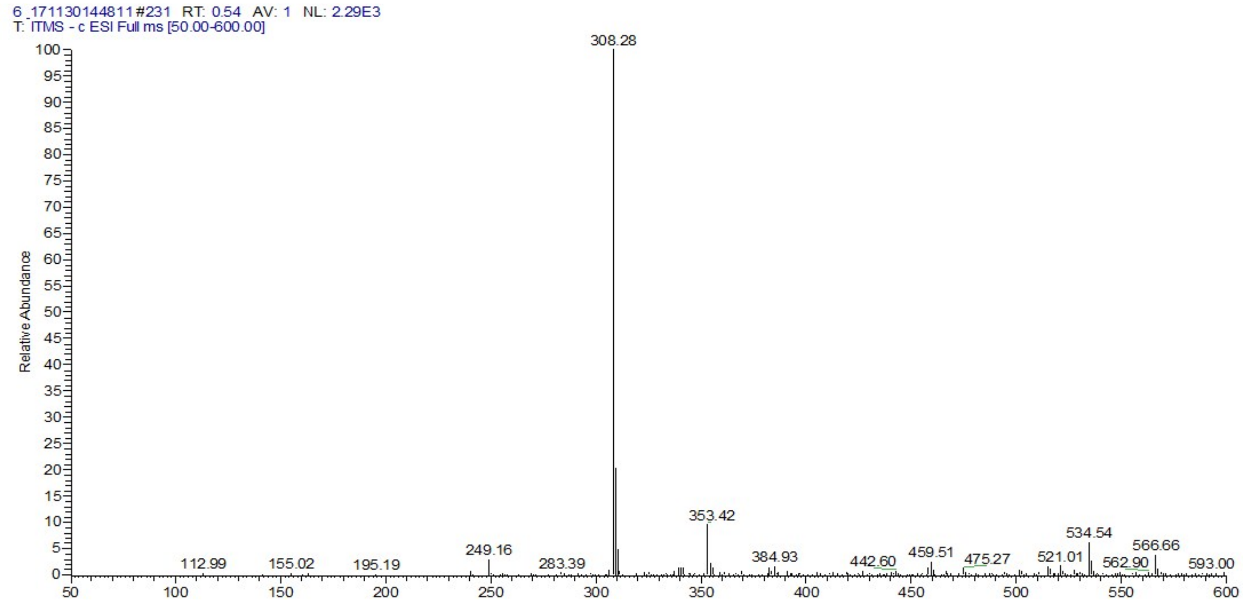

Figure 5 negative ESI-mass spectrum of 4-(prop-1-en-2-yl)-N-(3-(trifluoromethyl)phenyl)cyclohex-1-ene-1-carboxamide (**4**), [M-H] peak obtained on liquid- Mass spectrometer

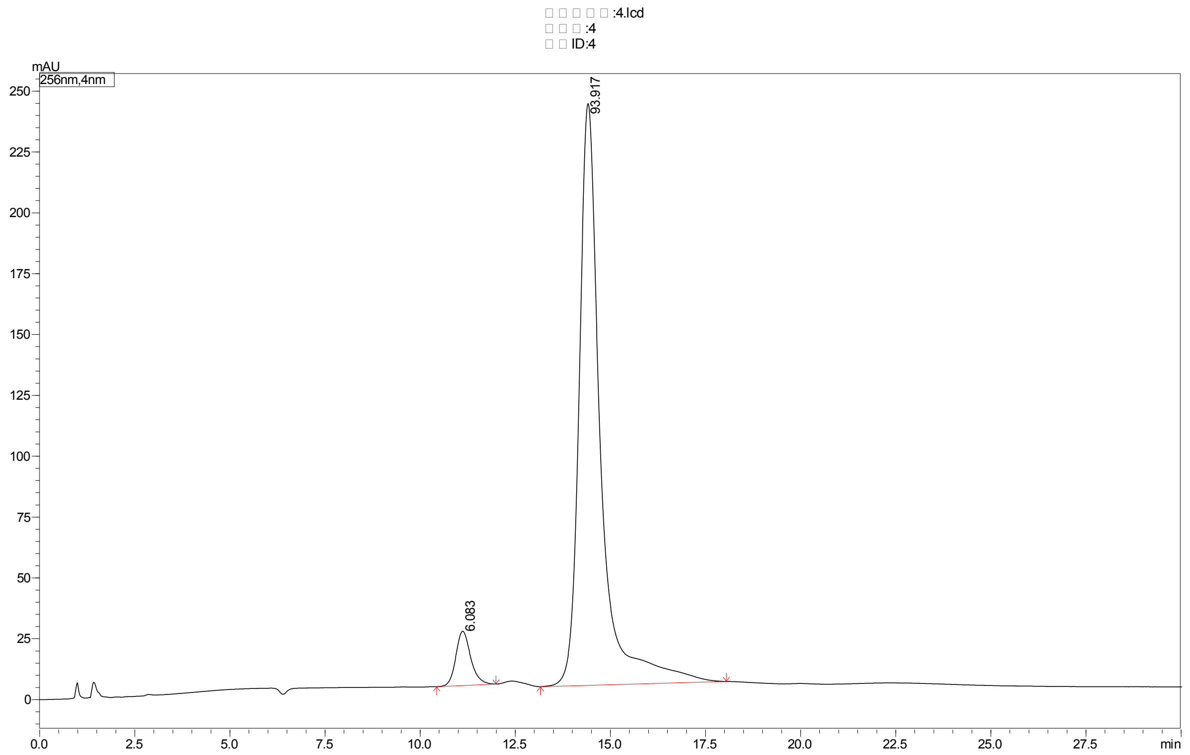

Figure S6 HPLC CHROMATOGRAPH OF 4-(prop-1-en-2-yl)-N-(3-(trifluoromethyl)phenyl)cyclohex-1-ene-1-carboxamide (**4**) obtained on a Schimazu Hplc system (conditions: Λmax= 256.0 nm, mobile phase methanol/double-deionized water= 70/30, flow rate = 1.0 ml, temperature= 30 ^{o}C, analysis time = 0-30 mins)

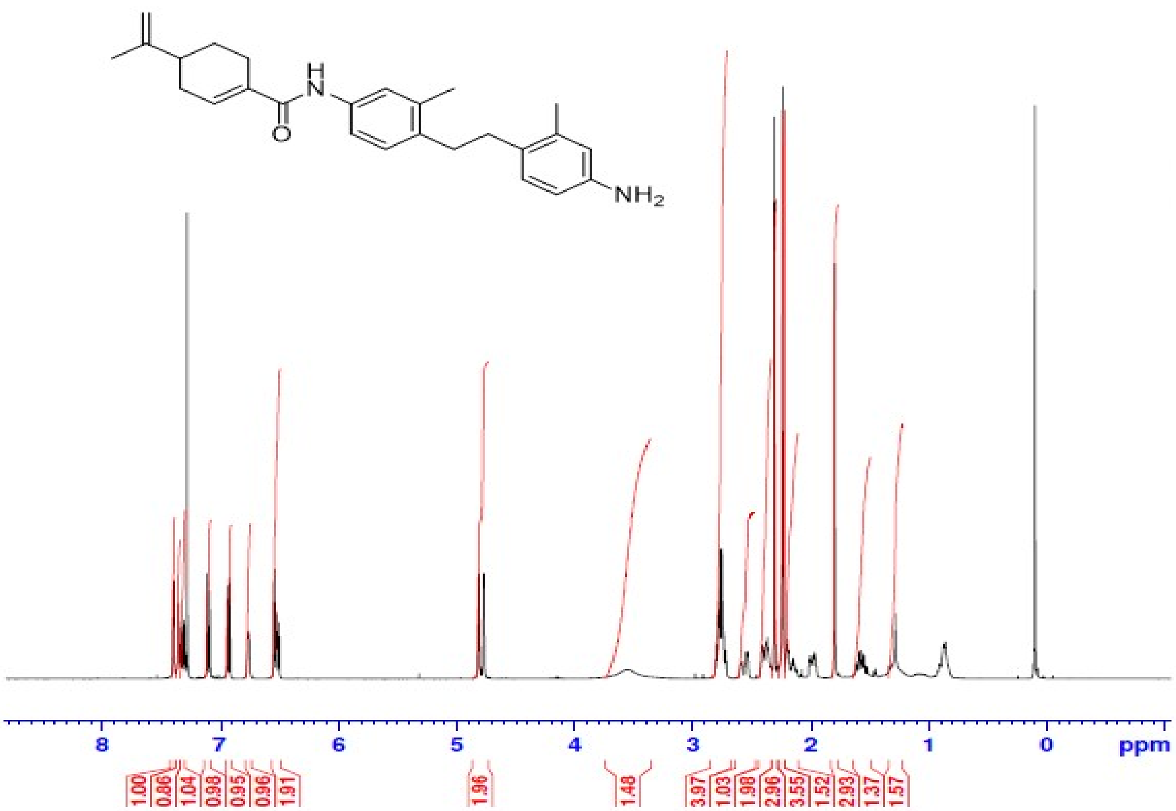

Figure S7 ^{1}H NMR spectrum of N-(4-(4-amino-2-methylphenethyl)-3-methylphenyl)-4-(prop-1-en-2-yl)cyclohex-1-ene-1-carboxamide (**5**) obtained on NMR spectrometer (Bruker 400 Ultra Shield™)

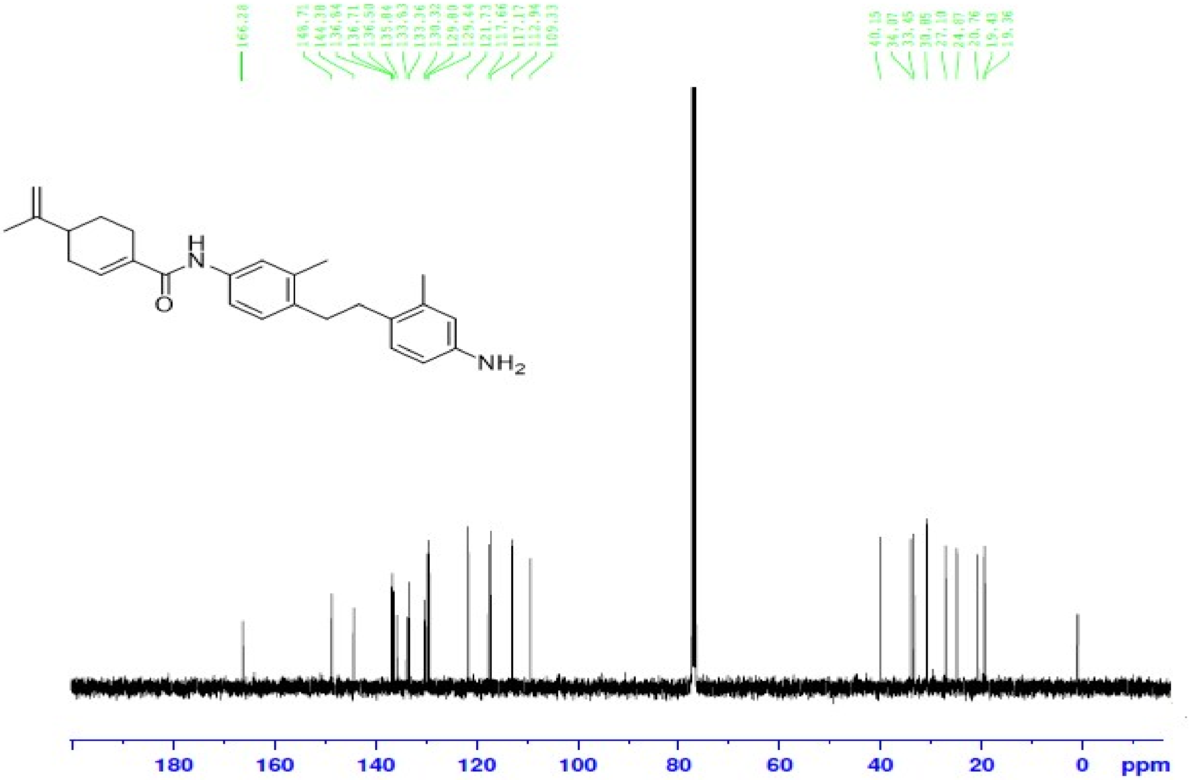

Figure S8 ^{13}C NMR spectrum of N-(4-(4-amino-2-methylphenethyl)-3-methylphenyl)-4-(prop-1-en-2-yl)cyclohex-1-ene-1-carboxamide (**5**) obtained on NMR spectrometer (Bruker 400 Ultra Shield^{TM})

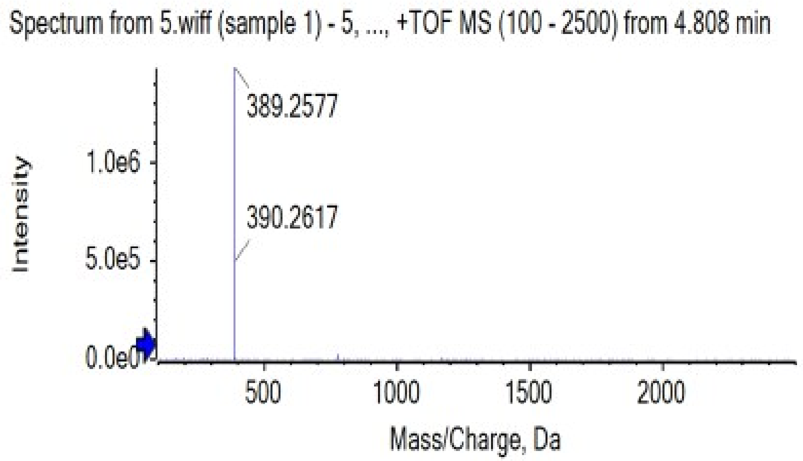

Figure S9 HRMS OF N-(4-(4-amino-2-methylphenethyl)-3-methylphenyl)-4-(prop-1-en-2-yl)cyclohex-1-ene-1-carboxamide (**5**) [M+H] peak

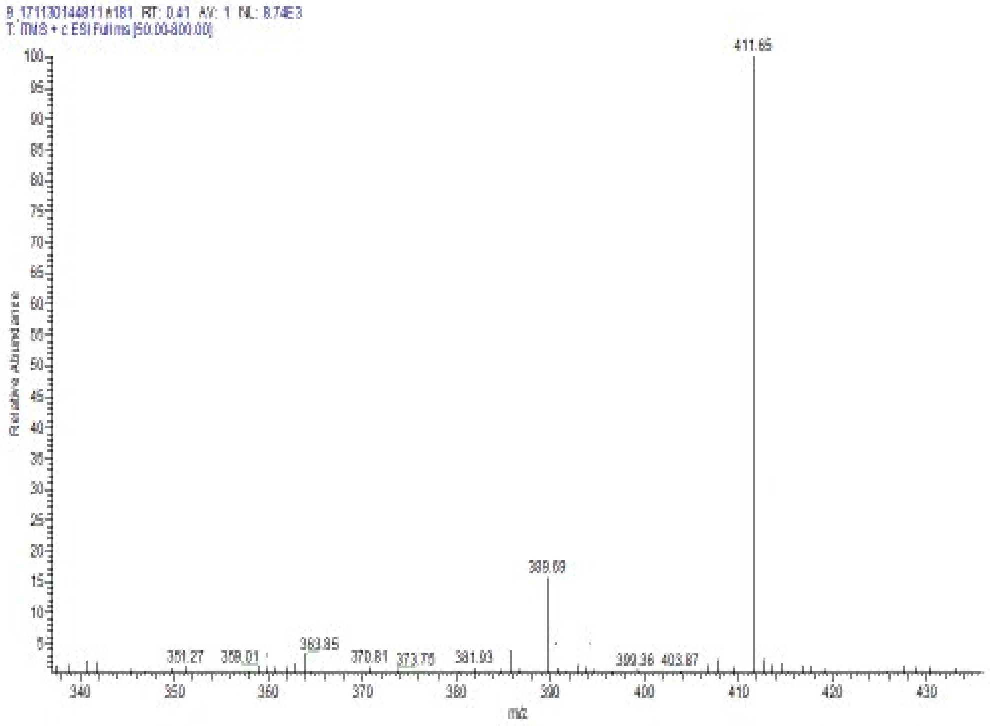

Figure S10 ESI-mass spectrum of N-(4-(4-amino-2-methylphenethyl)-3-methylphenyl)-4-(prop-1-en-2-yl)cyclohex-1-ene-1-carboxamide (5), [M+H] and [M+Na] peaks obtained on liquid- mass spectrometer

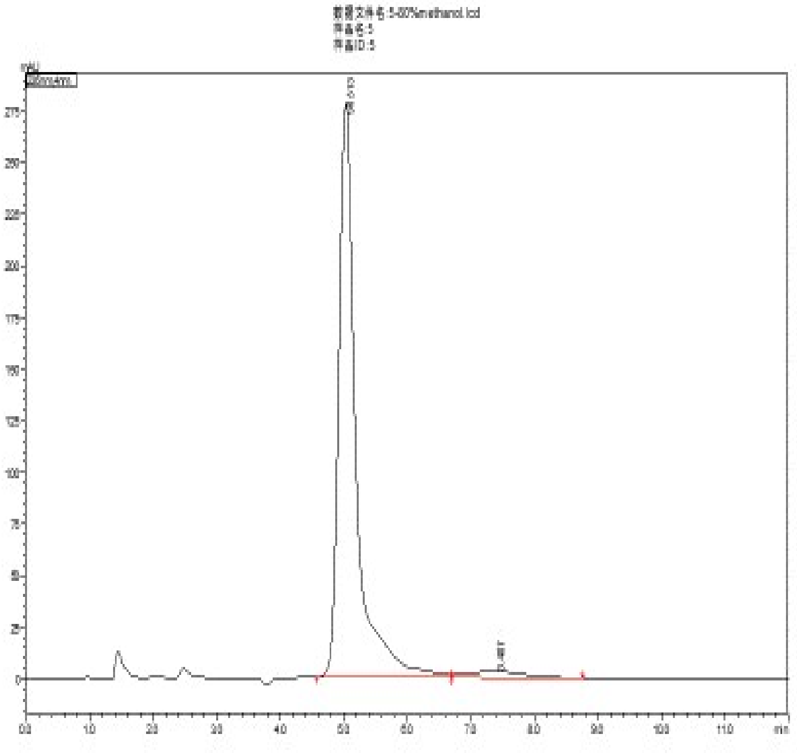

Figure S11 HPLC chromatograph of N-(4-(4-amino-2-methylphenethyl)-3-methylphenyl)-4-(prop-1-en-2-yl)cyclohex-1-ene-1-carboxamide (**5**) obtained on a Schimazu Hplc system (conditions: Λmax= 236.0 nm, mobile phase methanol/double-deionized water= 80/20, flow rate = 1.0 ml, temperature= 30 ^{o}C, analysis time = 0-20 mins)

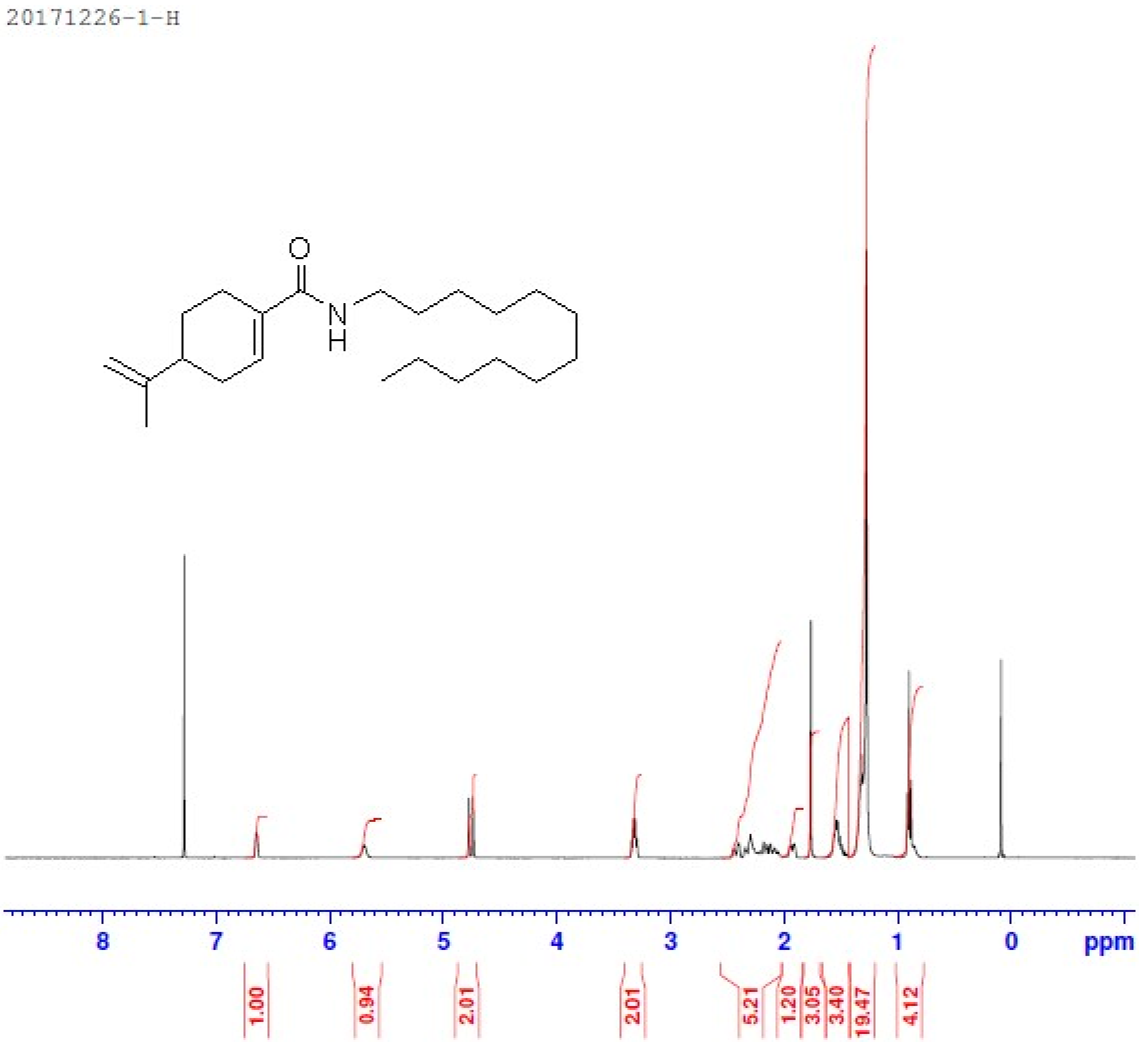

Figure S12 ^{1}HNMR spectrum of N-dodecyl-4-(prop-1-en-2-yl)cyclohex-1-ene-1-carboxamide (**2**) obtained on NMR spectrometer (Bruker 400 Ultra Shield^{TM})

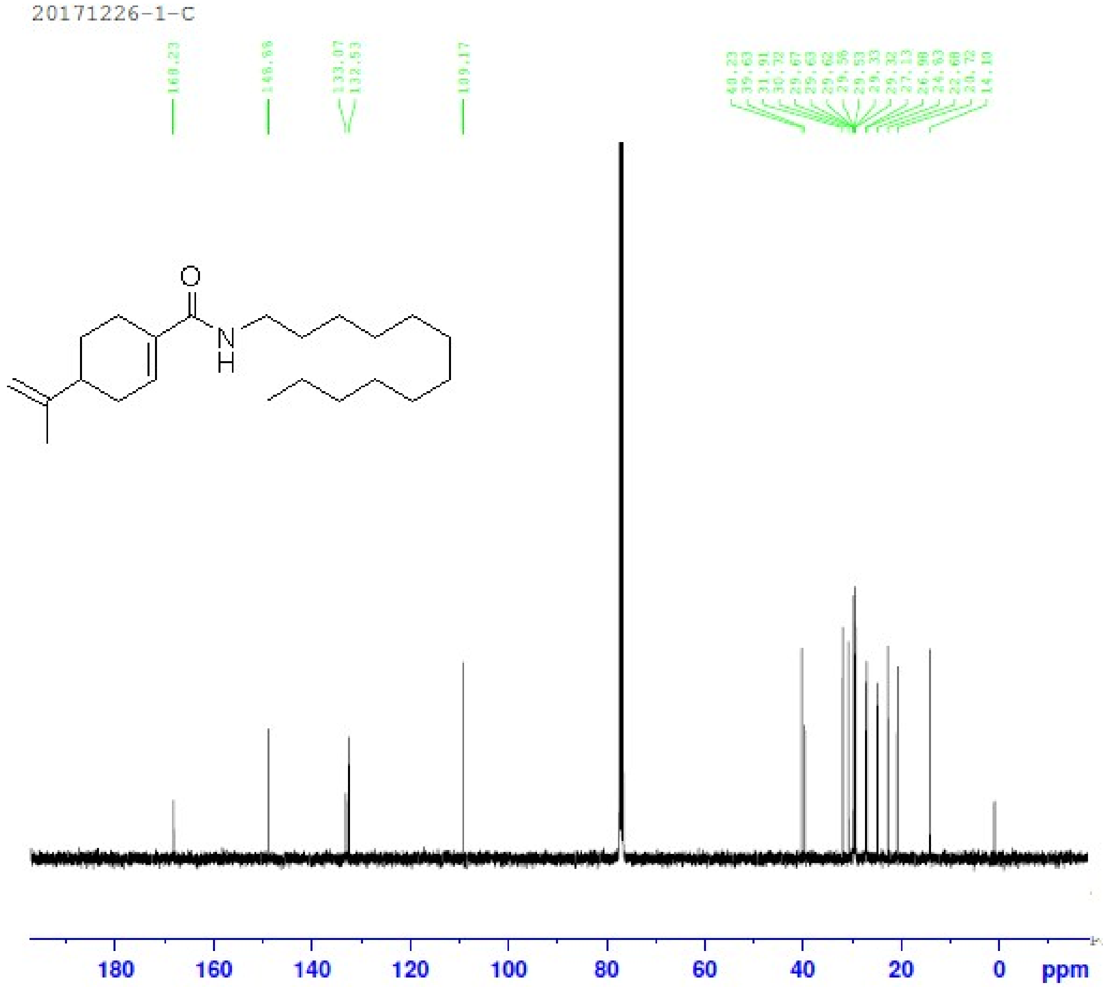

Figure S13 ^{13}CNMR spectrum of N-dodecyl-4-(prop-1-en-2-yl)cyclohex-1-ene-1-carboxamide (**2**) obtained on NMR spectrometer (Bruker 400 Ultra Shield^{TM})

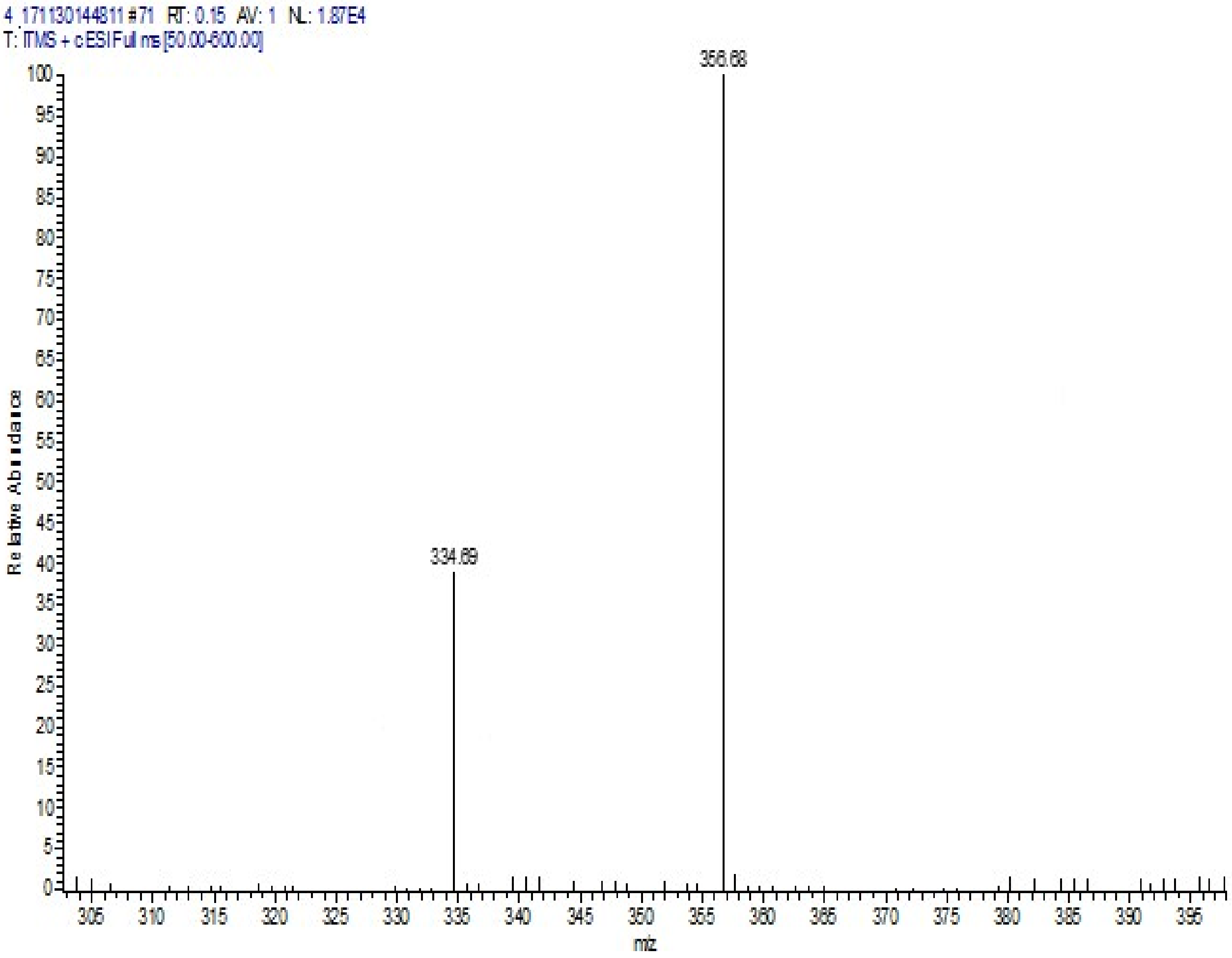

Figure S14 ESI mass spectrum of N-dodecyl-4-(prop-1-en-2-yl)cyclohex-1-ene-1-carboxamide (2), [M+H] and [M+Na] peaks obtained on liquid- mass spectrometer

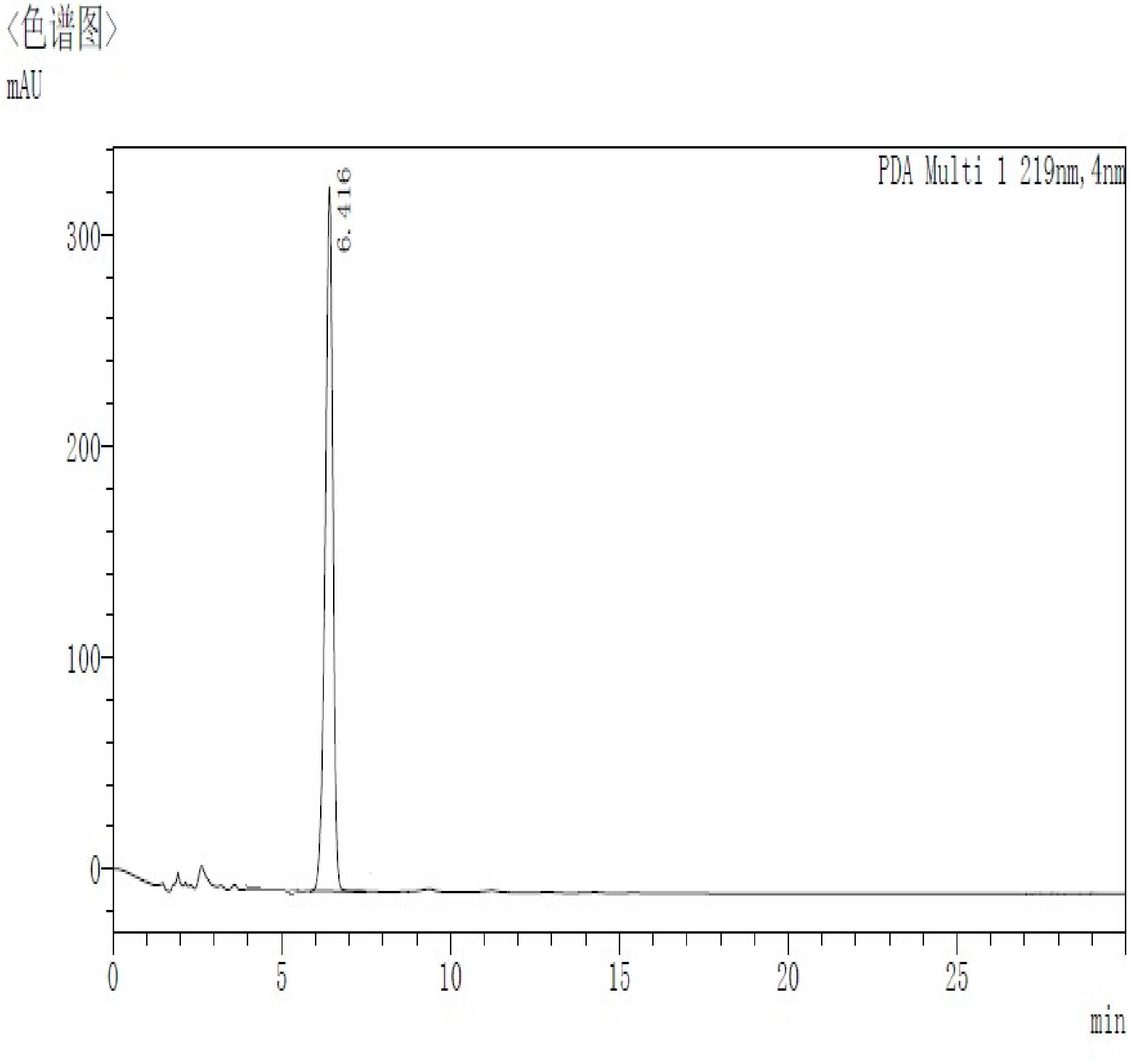

Figure 15S HPLC chromatograph of N-dodecyl-4-(prop-1-en-2-yl)cyclohex-1-ene-1-carboxamide (2) obtained on a Schimazu Hplc system (conditions: Λmax= 219.0 nm, mobile phase methanol/double-deionized water= 90/10, flow rate = 1.0 ml, temperature= 30 ^{o}C, analysis time = 0-30 mins)
